# Identification of Potent VEGF Inhibitors for the Clinical Treatment of Glioblastoma, A Virtual Screening Approach

**DOI:** 10.31557/APJCP.2019.20.9.2681

**Published:** 2019

**Authors:** Mohini Yadav, Ravina Khandelwal, Urvy Mudgal, Sivaraj Srinitha, Natasha KhandekaR, Anuraj Nayarisseri, Sugunakar Vuree, Sanjeev Kumar Singh

**Affiliations:** 1 *In silico Research Laboratory, Eminent Biosciences,*; 2 *Bioinformatics Research Laboratory, LeGene Biosciences Pvt Ltd., Indore – 452 010, Madhya Pradesh, *; 3 *Department of Biotechnology, Lovely Faculty of Technology and Sciences, Division of Research and Development, Lovely Professional University, Phagwara, Punjab-144411,*; 4 *Computer Aided Drug Designing and Molecular Modeling Lab, Department of Bioinformatics, Alagappa University, Karaikudi-630 003, Tamil Nadu, India.*

**Keywords:** VEGF, glioblastoma, virtual screening, molecular docking, ADMET profiling, drug likeness verification

## Abstract

Vascular endothelial growth factor (*VEGF*) expression could be found in all glioblastomas. *VEGF* takes part in numerous changes including the endothelial cell proliferation, the vasculature of solid tumor: its survival invasion, and migration, chemotaxis of bone marrow-derived progenitor cells, vasodilation and vascular permeability. *VEGF* inhibition can be a smart therapeutic strategy because it is extremely specific and less toxic than cytotoxic therapy. To establish better inhibition of *VEGF* than the current inhibitors, present study approach is by molecular docking, virtual screening to illustrate the inhibitor with superior affinity against *VEGF* to have a cautious pharma profile. To retrieve the best established and high-affinity high affinity molecule, Molegro Virtual Docker software was executed. The high-affinity scoring compounds were subjected to further similarity search to retrieve the drugs with similar properties from pubchem database. The completion of virtual screening reveals that PubChem compound SCHEMBL1250485 (PubChem CID: 66965667) has the highest affinity. The study of the drug-likeness was verified using OSIRIS Property Explorer software which supported the virtual screened result. Further ADMET study and drug comparative study strongly prove the superiority of the new established inhibitor with lesser rerank score and toxicity. Overall, the new inhibitor has higher potential to stop the expression of *VEGF* in glioblastoma and positively can be further analysed through In vitro studies.

## Introduction

The term ‘glioma’ comprehends all tumors encompassing the glial cell origin, including astrocytoma grades I, II, III and IV (Schwartzbaum et al., 2006). Unfortunately, grade 4 which is Glioblastoma multiforme (GBM), is the most aggressive and also the most common in humans. This brain tumor drew the noteworthy attention of scientists and doctors all over the world as patients with GBM die within a year having no scope of long term survival. However, the research evaded progressively high and complex attempts at therapy over the past half-century (Holland, 2000) which still accounts for 12 percent to 15 percent of all intracranial tumors and 50 to 60 percentage of astrocytic tumors with an annual incidence rate of 5.26 per 100,000 population new diagnoses per year (Rosell et al., 2008, Omuro and DeAngelis, 2013). The survival rate is almost similar in various patients and also not influenced by the patient’s sex, the grade of tumor and the extent of surgery (Simpson and Platts, 1976). But the patients diagnosed with GBM in the early ages of 40 to 50 years show higher 5-year overall survival rates (Stupp et al., 2009). Glioblastoma shows multiple complex character types as its name has multiple forms which is one of the reasons for the resistance of GBM to therapeutic intervention. GBM is excessively showing regions of necrosis and haemorrhage (Holland, 2000). This lethal GBM reappears in nearly all patients and no effective generalized treatment exists for the recurrent diseases up till now. Therefore, advances in the GBM treatment and drugs in all clinical and scientific aspects are urgently needed (Lee et al., 2017). A case study of 153 recurrent glioblastoma patients in 2014 summarises the approval by the FDA on the most effective drug bevacizumab, which can increase the survival up tomonths, which 38% and combined treatment of bevacizumab with lomustine can increase the survival rate up to 59%. This data indicates the need for new and more effective drugs and treatment options for the patients of recurrent GBM (Mehta and Brem et al., 2014). Glioblastoma multiform has two noticeable features that are angiogenesis and tumorigenesis. One a vascular factor that can connect both the noticeable features (Greenberg et al., 2005). Angiogenesis controls the growth and synthesis of all the tumors by forming new blood vessels from pre-existing vessels (Folkman, 1971). Tumor angiogenesis is coordinated by a synchronized increase in the expression of genes, including *VEGF*,* IL-8* and *IL-6*, acidic as well as basic fibroblast growth factor (FGF), angiopoietins and the hypoxia-inducible factor 1 alpha (HIF-1α), with downregulation of endogenous angiogenesis inhibitors such as: endostatin, thrombospondins, interferon and angiostatin (Hanahan and Folkman, 1996). *VEGF* is a supreme common denominator required for tumor angiogenesis and pathogenesis (Ferrara et al., 2003).

The *VEGF* and its receptors are expressed in GBM and angiogenesis are involved in nearly every stage of cancer, from the first stage of cancer formation to the last stage of distant metastasis. *VEGF* inhibition can be a smart therapeutic strategy because it is extremely specific and less toxic than cytotoxic therapy. *VEGF *inhibitors offer a means to control a heterogeneous tumor population by influencing a relatively homogeneous endothelial population. Therefore the current study ended with the better inhibitor for *VEGF* by using docking and drug designing to ascertain that the new compound plays a significant role in upsurge the survival of the GBM patients.

## Materials and Methods

*Selection of inhibitor*

Innitiating the inhibitor selection, existing inhibitors of *VEGF* that target GBM were selected from several literatures. The availability of total numbers of established inhibitors were 16, selected for further observations. Some inhibitors were lacking their 3D structures. The 3D structures of all those compounds were modelled using Marvin Sketch and was saved in 3D SDF format (Ali et al., 2019; Khandelwal et al., 2018). All the 16 inhibitors accordingly having Pubchem ID with 3D structures and prepared 3D structures are present in the Table1.

*Protein and Ligand preparation*

The crystal structure of the target protein, an extracellular domain of *VEGF* was retrieved from protein Data Bank (PDB) with PDB ID: 3V2A (Brozzo et al., 2012). Few amino acids side-chain atoms were missing in the receptor structure 3V2A. A reconstruction of the whole side-chain was performed using Modellar software. 21 side-chain atoms were added in the present 3D structure and the energy minimization was performed in vacuo with the Gromos 96 algorithm using 43B1 parameters set, without reaction field (Akare et al., 2014; Babitha et al., 2018; Bandaru et al., 2013; Bandaru et al., 2014). After adding the missing side-chain atoms the energy was -11310.358 KJ/Mol which shows better stability to the 3D structure of *VEGF*. Hence the same was used for the molecular docking studies. The inhibitors accomplishing a Pubchem ID were retrieved the 3D conformer of inhibitors. Further preparation of ligand was preceded by taking the 3D structure of all those compound embedded in LegPrep module, an application of Schrodinger suite (Schrodinger. LLC, New York, NY) and were optimized through OPLS 2005 force field algorithm (Bandaru et al., 2015a; Bandaru S et al., 2015b; Babitha et al., 2015; Chandrakar et al., 2013; Divya Jain et al., 2019; Dunna et al., 2015a; Gudala, et al., 2015; Majhi et al., 2018). The prepared ligands were saved in a single SDF file for further docking studies (Bandaru et al., 2016; Basak et al., 2016a; Dunna et al., 2015b; Mendonça-Junior et al., 2019; Nasr et al., 2015).

*Molecular docking*

The molecular docking study was performed by using Molegro Virtual Docker (MVD) which is unified with high potential Piece Wise Linear Potential (PLP) and MVD scoring function (Bandaru et al., 2017a; Basak et al., 2016b; Gutlapalli et al., 2015; Kelotra et al., 2014; Natchimuthu et al., 2016; Nayarisseri et al., 2018; Sharda et al., 2019; Padmini et al., 2019). The pre-prepared 16 ligands were saved in one single SDF file. PDB file of target protein consisting of pre-existing ligands were removed. It was then prepared further by detecting cavities. A cavity, namely the fourth cavity bearing a volume of 13.825Ao was targeted for further procedure of docking with ligands. Docking process possessed the of maximum iteration of 1,500, maximum population size 50, Grid solution 0.2; having a binding affinity, the protein and ligands were evaluated on the following conformation of the Internal Electrostatic interaction (Internal ES), sp2-sp2 torsions, and internal hydrogen bond interaction. Binding site defined the first cavity according to the highest volume. The post dock study involved energy minimization and H-bond optimization. After docking, to minimize the complex energy of ligand-receptor interaction, the Nelder Mead Simplex Minimization (using non grid force field and H-bond directionality) was used (Sahila et al., 2017; Bandaru et al., 2017b; Kelotra et al., 2014; Monteiro et al., 2018; Nasr et al., 2015).

*Virtual screening*

With reference to our target query compound Cediranib, a similarity search was executed to obtain the best compound having a greater affinity other than any established drugs against the PubChem database developed by NIH, which is one of the public chemical repository containing 93 million chemical compounds database(Sinha et al., 2014; Sinha et al., 2018; Trishang et al., 2019; Vuree et al., 2013; Nayarisseri et al., 2019; Sharma et al., 2018; Shaheen et al., 2015; Shameer et al., 2017). The filtrations property parameter set by component rule of Lipinski’s rule of five at threshold >=95% were done against NCBI’s PubChem compound database (Nayarisseri et al., 2015; Sharda et al., 2017; Khandelwal et al., 2018; Khandekar et al., 2016; Palak S et al., 2019; Patidar K et al., 2019; Patidar, K et al., 2016; Sinha et al., 2015; Sahila et al., 2015). These compounds were with the same procedure, preceded for Molecular Docking with the target protein *VEGF* to find the compound having surpassed affinity.

*Drug – Drug comparative study*

The unnamed complex structure was retrieved from the established drug docking result. It was cleaned by removing all the ligands, constraints, and cavities except the protein which is eventually imported with the best posed inhibitor and exported as best drug docked file in SDF format (Praseetha et al., 2016a; Praseetha et al., 2016b; Rao et al., 2010). The complex structure was retrieved from the virtual docking result and the procedure was repeated. The excel sheet was prepared to check all the affinities, hydrogen interactions, steric energy and lowest re-rank score to identify the best inhibitor. 

*ADMET studies*

The admetSAR database provides a free interface to query a distinct biological and chemical profile. The properties of ADMET profile include Adsorption, Digestion, Metabolism, Excretion, Toxicity which perform key roles in the development and discovery ofdrugs (Nayarisseri et al., 2019b; Sweta et al., 2019; Aher et al., 2019). The database preferably comprises of the 5 quantitative regression models and 22 qualitative classifications which provides the result with highly predictive precision. This estimation over properties of this database was predicted using admetSAR (http://lmmd.ecust.edu.cn:8000/). With the superior affinity of best docked virtual screened compound SCHEMBL1250485 (PubChem CID:66965667) and best-established drug Cediranib(AZD2171) PubChem CID:9933475, the bioactivity properties and toxicity was predicted by using admetSAR (Cheng et al., 2012).

*Drug-Likeness Prediction Studies*

A chemical inhibitor can be an effective drug if it absorbed in the required time and distributed throughout the system before getting filtered by the excretory system. In silico drug-likeness study along with ADMET has done by OSIRIS Property Explorer, that presents a group of effective parameters which help in accelerating the discovery of more effective targets and ultimately leading to drugs with the predicted biological activity which saves time and expenses (Nayarisseri et al., 2018; Sharda et al., 2019).

*Web servers, Software and Suites Used*

All the chemical 3D structures were retrieved from NCBI’s Pubchem in SDF format and lacking 3D structure was drawn in Marvin Sketch 5.6.0.2, (1998-2011, Copyright^©^ChemAxonLtd). The ligands were optimized by using the software Schrodinger suite 2013 (Schrodinger.LLC, 2009, New York, NY). The flexible Docking was performed by taking target and all the compounds in Molegro Virtual Docker 2010.4.0.0.Molecular Visualization was done with Accelrys Discovery Studio® Visualizer 3.5.0.12158(Copyright^©^ 2005-12, Accelrys Software Inc.). ADMET profiles were studied and calculated using admetSAR (Laboratory of Molecular Modeling and Design. Copyright^©^ 2012 East China University of Science and Technology, Shanghai Key Laboratory for New Drug Design).

## Results

*Docking results*

The docking studies of complete pre-established 16 drugs resulted in Cediranib (AZD2171) (PubChem ID: 9933475) as the best-established compound (Table 2). Cediranib (AZD2171) shows the higher affinity score directed towards our target protein and has the great affinity properties as molecular weight 450.514 g/mol, hydrogen bond donor count 1 and hydrogen bond acceptor count 7, topological polar surface area 72.5 A^2^ and logP value is 4.9. Thus the compound Cediranib has superior inhibitory affinity over protein VEGF in Glioblastoma.

*Virtual screened results*

**Figure 1:**
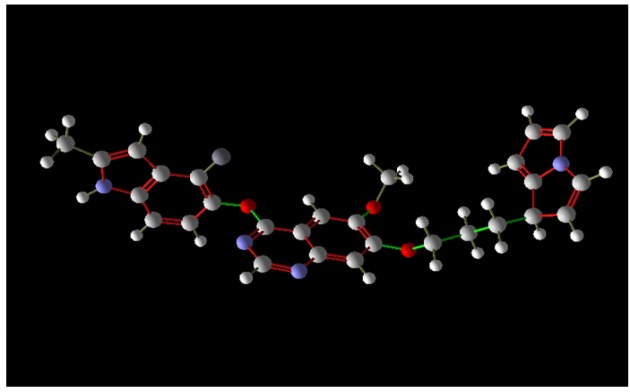
Best Virtual Screened Compound SCHEMBL1250485 (PubChem CID:66965667) 3D Structure Obtained from Pubchem

Advance similarity search for the compound cediranib displayed 139 compounds. Table 3 shows the ten superior docking result of entire 139 virtual screened compounds showed SCHEMBL1250485 (PubChem CID:66965667) shown in (Figure 1) as a high affinity compound with the lowest rerank score. This compound has a molecular weight of 484.531 g/mol, 1 hydrogen bond donor, 6 hydrogen bond acceptor, a topological surface area of 74.2 A^2^ and a log P value is 5.5. Among all these 139 compounds, the drug with PubChem CID:66965667 has much potential inhibition against Glioblastoma over the target protein VEGF.

*Drug-Drug comparison*

Table 3 discloses the re-rank scores of the compounds against the target protein *VEGF* on Glioblastoma. (Table 4), the total energy of the newly found inhibitor Pubchem ID- 66965667 was lowest among the entire virtual screened compound shows its better affinity. Interestingly, the other interaction of both the compounds displaying the virtual screened compound has less affinity interaction properties according to the steric energy of PLP (Piecewise Linear Potential) but steric energy of LJ12-6 (Leonard-Jones approximation) is almost same as the pre-established Cediranib. Whereas the hydrogen bonds stability is seen more in the virtual screened drug than established inhibitor Cediranib. So-and-so, it demonstrates that the virtual screened compounds have higher potential inhibition towards the target protein *VEGF* for Glioblastoma.

*Pharmacophore mapping*

Pharmacophore mapping provides a three- dimensional essential systematic topographies of molecular interaction with specific target receptors apart from the method of molecular docking. Pharmacophore studies provide an accurate query on the optimum interaction with suitable target annotations and represent the aligned poses of the molecule and help us to find the high interaction mode between the target proteinand the new compound. In spite of admirable affinity and good interaction profile of inhibitor Cediranib, Pubchem CID: 9933475 proves to have better screened result, which carries forward the study to the Pharmacophore results. Pharmacophore mapping also showed the positive intensities of electrostatics and intensities that vary in hydrophobicity, aromatic, ionizability and the distribution of interpolated charges respectively.

**Figure 2:**
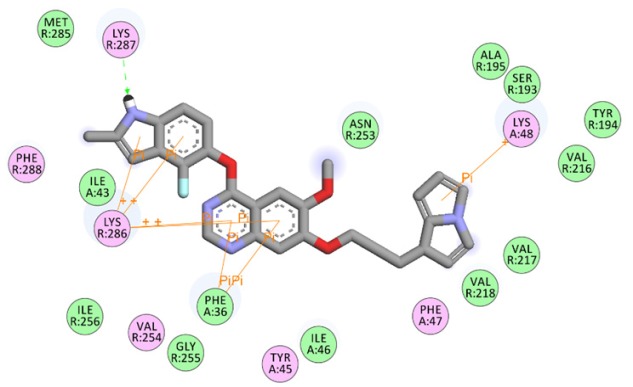
Screened Compound SCHEMBL1250485 (CID:66965667) Showing the Van Der Waal Interactions

The residue interaction of virtually screened chemical compound SCHEMBL1250485 (Pub CID: 66965667) in the cavity of target protein VEGF (Figure 2) displayed residues with ligands where the green circled are van der Waals interaction and the pink residues circled are Electrostatic interactions. A green arrow between residues and ligand shows Hydrogen bond interaction. The virtual compound has three hydrogen bonds among which one green dotted arrow shows LYS R287 as hydrogen donor in Figure 2. Subsequently, green circles and brown lines represent the van der Waal interaction and pi-pi interactions respectively. It was clear that the compound showed high Van der Waal interaction with Met R-285, Ile A-43, Ile R-256, Ile A-46, Gly R-255, Phe A-36, Asn R-253, Val R-218, Val R-217, Val R-216, Ala R-195, Ser R-193, and Tyr R-194. The compound also displays the pi-pi interaction with Lys R-286, Phe A-36, and Lys A-48. 

**Figure 3:**
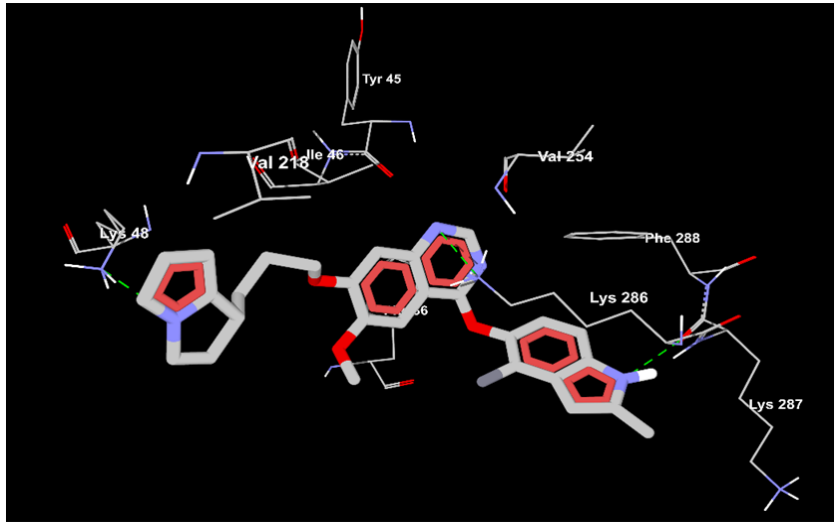
Screened Compound SCHEMBL1250485 (CID:66965667) Having the Hydrogen Bond Interaction

In Figure 3 virtual screened SCHEMBL1250485 (CID:66965667) has three hydrogen bonds with Lys 48, Lys 287 and Lys 286 which are depicted by green dotted lines delineating the high affinity towards the active site of VEGF as compared to the established compound Cediranib (CID:9933475) which has zero hydrogen bond.

**Figure 4:**
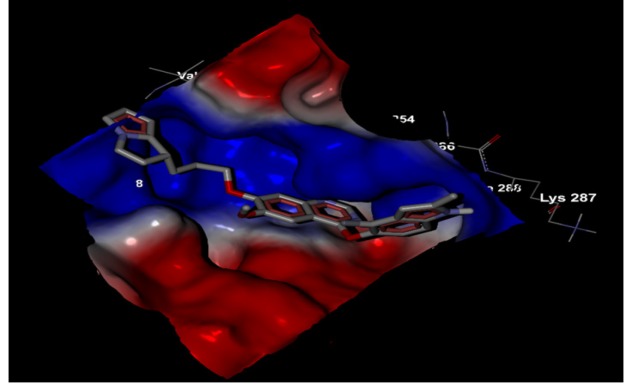
Screened Compound SCHEMBL1250485 (CID:66965667) Showing Electrostatic Interaction

**Figure 5:**
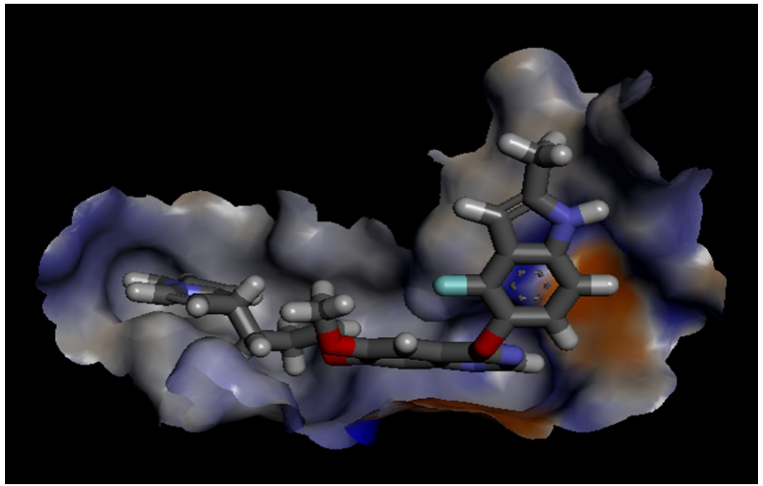
Screened Compound SCHEMBL1250485 (CID:66965667) Revealed the Aromatic Interaction

The electrostatic surface of the protein has two types of shaded area, the blue shaded region being the electropositive surface and the red shaded region representing the electronegative surface. The virtual screened compound SCHEMBL1250485 (CID:66965667) embedded in the VEGF protein cavity with a high-affinity shown in Figure 4. The chemical compound SCHEMBL1250485 (CID:66965667) was completely sheltered in the highly electropositive residues shown in the blue colour. Figure 5 depicts the aromatic interaction of target protein VEGF cavity against screened compound SCHEMBL1250485 (CID:66965667). The targeted protein VEGF displays the aromatic edge and face by the shades of blue and orange respectively. The molecule was showing less aromatic interaction.

*ADMET profile*

In Table 5, the ADMET prediction of both the best-screened compound SCHEMBL1250485 (CID:66965667) and established Cediranib (AZD2171) CID:9933475, is nearly equivalent and done with admetSAR tool. As accordingly brain penetration prediction that is BBB (Blood –Brain Barrier), cediranib displays the positive to the property of absorbing. Human Intestinal Absorption (HIA) of new drug shows a greater absorption in the intestine as the screened compound SCHEMBL1250485 (CID:66965667) as shown to have a positive tendency. The predictions on P-glycoprotein Substrate and P-glycoprotein Inhibition, Cediranib shows at a higher extent than the screened compound SCHEMBL1250485 (CID:66965667). The absorption site for the P-glycoprotein substrate for the screened compound SCHEMBL1250485 (CID:66965667) reveals it has less probability than established Cediranib (CID:9933475) as similarly P-glycoprotein Inhibitor shows the values with low probability. 

In addition to the distributionof subcellular localization in both the established and screened compounds in the mitochondria, the screened compound (CID:66965667) shows the distribution to have a high probability than others. In the case of metabolism, they vary in some points like CYP450 3A4, CYP450 2C19 Inhibitor, and CYP450 1A2 Inhibitor where both the compounds are acting as substrate as well as the inhibitors. CYP450 2C9 acts as non-inhibitor for cediranib. Overall both the compounds display the equivalent high inhibitory effect towards the target protein. Further study of bioactivity in the profile of excretion and toxicity is almost equivalent, but in reference to Carcinogens they vary as the virtual screened compound is shown to have high amount of non-carcinogens than established docked compound. The compounds are mutagenic or not that can predict by ADMET regression toxicity study. Both the compounds in the properties of Rat Acute Toxicity are nearly equal to each other. But the possibility of having higher toxicity than these two molecules is shown in (Table 6). Additional study of bioactivity in the profile of excretion and toxicity shows it to be almost equivalent, but in reference to fish toxicity, they vary as Cediranib shows high toxicity than the virtual screened compound.

**Table 1:** Established VEGF Inhibitors with Pubchem CID

SI	Inhibitor	Pub Id	Mw (g/mol)	HBD	HBA	XlogP	Reference
1	Semaxanib (SU5416)	5329098	238.29	2	1	2.5	(Vajkoczy et al., 1999)
2	Vatalanib	151194	346.818	1	4	4.5	(Gerstner et al.,2009)
3	Sunitinib	5329102	398.482	3	4	2.6	(Gerstner et al.,2009)
4	Sorafenib	216239	464.829	3	7	4.1	(Gerstner et al.,2009, Loges)
5	Cediranib(AZD2171)	9933475	450.514	1	7	4.9	(Batchelor et al., 2007a; Gerstner et al.,2009)
6	CEP-7055	9936664	525.649	1	5	4.5	(Jones-Bolin et al., 2006a)
7	XL-184	25102847	501.514	2	7	5.4	(Gerstner et al.,2009; Zhang et al., 2010; Hernandez-Pedro et al., 2013)
8	Nelfinavir	64143	567.789	4	6	5.7	(Kast et al., 2013; Mangraviti et al., 2017)
9	AEE788	10297043	440.595	2	5	4.6	(Gerstner et al., 2009; Reardon et al., 2012)
10	Amprenavir	65016	505.63	3	8	2.9	(Pore et al., 2006)
11	Acriflavine	443101	468.989	4	6	--	(Mangraviti et al., 2017)
12	Pazopanib	10113978	437.522	2	8	3.1	(Gerstner et al., 2009; Taylor and Gerstner, 2013)
13	Vandetanib or ZD6474	3081361	475.362	1	7	4.9	(Gerstner et al., 2009; Taylor and Gerstner, 2013; Jawhari et al., 2016)
14	Pegdinetanib or CT322	86278317	694.754	6	15	-5.7	(Mamluk et al., 2010)
15	Sodium butyrate	5222465	110.088	0	2	--	(Sawa et al., 2002)
16	Trichostatin A	444732	302.374	2	4	2.7	(Sawa et al., 2002)

**Table 2:** Established Drugs Docking Result

Ligand PubChem ID	MolDock Score	Rerank Score	H-Bond	MW
9933475	-137.596	-108.652	-3.00765	450.505
9933475	-133.384	-106.781	-2.68048	450.505
9933475	-136.24	-102.165	-2.64373	450.505
9936664	-148.731	-101.716	-2.97578	525.638
10297043	-121.812	-93.5305	0.000	440.583
5329102	-128.833	-93.4814	-0.58886	398.474
10297043	-124.403	-92.0401	-0.14425	440.583
5329102	-129.038	-90.9477	-4.07277	398.474
25102847	-106.939	-88.7836	-3.54584	501.506

**Table 3:** Virtual Screened Compounds Docking Result with Reference to High Affinity Cediranib (AZD2171)

Ligand	MolDock Score	Rerank Score	H-Bond	MW
66965667	-152.508	-117.928	-4.89163	484.522
66965667	-147.28	-115.559	0.0000	484.522
69090654	-141.166	-114.958	-3.79463	478.515
69090654	-141.166	-114.958	-3.79463	478.515
10196058	-138.069	-113.024	-4.18063	511.564
44156780	-138.201	-112.568	-3.52407	450.505
44156780	-138.201	-112.568	-3.52407	450.505
44156354	-140.988	-110.062	-2.16748	450.505
44156354	-140.988	-110.062	-2.16748	450.505
1.23E+08	-129.443	-109.309	-3.53349	466.505

**Table 4:** Drug – Drug Comparative Study

	Virtual Screened (Pubchem CID: 66965667)	Established (Cediranib Pubchem CID: 9933475)
Energy overview:Descriptors	Rerank Score	Rerank Score
Total Energy	-117.927	-108.925
External Ligand interactions	-133.554	-126.948
Protein-Ligand interactions	-133.554	-126.948
Steric (by PLP)	-106.676	-103.941
Steric (by LJ12-6)	-23.001	-23.008
Hydrogen bonds	-3.876	0
Internal Ligand interactions	15.627	18.023
Torsional strain	5.386	2.851
Torsional strain (sp2-sp2)	0	0
Hydrogen bonds	0	0
Steric (by PLP)	0.369	1.738
Steric (by LJ12-6)	9.871	13.435
Electrostatic	0	0

**Table 5:** ADMET Profile Calculation of Both Best Docked Compound by AdmetSAR

Model	Virtual screened compound SCHEMBL1250485 (CID:66965667)		Cediranib (AZD2171) CID:9933475	
	Result	Probability	Result	Probability
Absorption				
Blood-Brain Barrier	BBB+	0.9202	BBB+	0.972
Human Intestinal Absorption	HIA+	0.993	HIA+	0.9928
Caco-2 Permeability	Caco2+	0.5552	Caco2+	0.5919
P-glycoprotein Substrate	Non-substrate	0.6185	Substrate	0.6286
P-glycoprotein Inhibitor	Inhibitor	0.5895	Inhibitor	0.7308
	Inhibitor	0.6986	Inhibitor	0.8118
Renal Organic Cation Transporter	Non-inhibitor	0.6097	Inhibitor	0.6936
Distribution				
Subcellular localization	Mitochondria	0.7723	Mitochondria	0.6861
Metabolism				
CYP450 2C9 Substrate	Non-substrate	0.7816	Non-substrate	0.8505
CYP450 2D6 Substrate	Non-substrate	0.7468	Non-substrate	0.5395
CYP450 3A4 Substrate	Substrate	0.7121	Substrate	0.7607
CYP450 1A2 Inhibitor	Inhibitor	0.8174	Inhibitor	0.6053
CYP450 2C9 Inhibitor	Inhibitor	0.5491	Non-inhibitor	0.5716
CYP450 2D6 Inhibitor	Non-inhibitor	0.7921	Non-inhibitor	0.6498
CYP450 2C19 Inhibitor	Inhibitor	0.7867	Inhibitor	0.7982
CYP450 3A4 Inhibitor	Inhibitor	0.5804	Inhibitor	0.6267
CYP Inhibitory Promiscuity	High CYP Inhibitory Promiscuity	0.9417	High CYP Inhibitory Promiscuity	0.922
Excretion Toxicity				
Human Ether-a-go-go-Related Gene Inhibition	Weak inhibitor	0.9458	Weak inhibitor	0.7652
	Non-inhibitor	0.5297	Inhibitor	0.8887
AMES Toxicity	Non AMES toxic	0.5649	Non AMES toxic	0.641
Carcinogens	Non-carcinogens	0.9335	Non-carcinogens	0.9197
Fish Toxicity	Low FHMT	0.5698	Low FHMT	0.545
Tetrahymena Pyriformis Toxicity	High TPT	0.9667	High TPT	0.8544
Honey Bee Toxicity	Low HBT	0.7818	Low HBT	0.8482
Biodegradation	Not ready biodegradable	1	Not ready biodegradable	1
Acute Oral Toxicity	III	0.6654	III	0.7212
Carcinogenicity (Three-class)	Non-required	0.4995	Non-required	0.6297

**Table 6:** ADMET Profile- Regression

		SCHEMBL1250485 (CID:66965667)	Cediranib(AZD2171) CID:9933475
Model	Unit	Value	Value
Absorption			
Aqueous solubility	LogS	-3.6396	-3.132
Caco-2 Permeability	LogPapp, cm/s	1.1549	0.972
Toxicity			
Rat Acute Toxicity	LD50, mol/kg	2.5448	2.5985
Fish Toxicity	pLC50, mg/L	0.8161	1.2558
Tetrahymena Pyriformis Toxicity	pIGC50, ug/L	0.6018	0.4099

**Table 7:** Comparative ADMET Profile with Two Test Ligands and Two Controls

Inhibitor name/Pub ID	BBB	HIA	CYP Substrate/inhibition	AMES toxicity	Carcinogenicity	LD50 in rat
66965667	0.9202	0.993	Nonsubstrate/inhibitor	0.5649	Non-carcinogens	2.5448
69090654	0.9401	0.9853	Nonsubstrate/noninhibitor	0.6817	Non-carcinogens	2.5589
Cediranib	0.972	0.9928	Nonsubstrate/inhibitor	0.641	Non-carcinogens	2.5985
9936664	0.842	0.9824	Nonsubstrate/noninhibitor	0.7183	Non-carcinogens	2.5968

**Table 8.** Drug-Likeness Prediction Through OSIRIS Property Explorer

S.N	Inhibitor	clogP	Log S	Molecular weight	TPSA
1	PubCID 66965667	5.92	-7.38	484	74.1
2	PubCID 69090654	4.61	-6.81	478	89.57
3	Cediranib	4.5	-6.56	450	72.5
4	PubID9936664	4.24	-6.25	525	72.8

**Figure 6:**
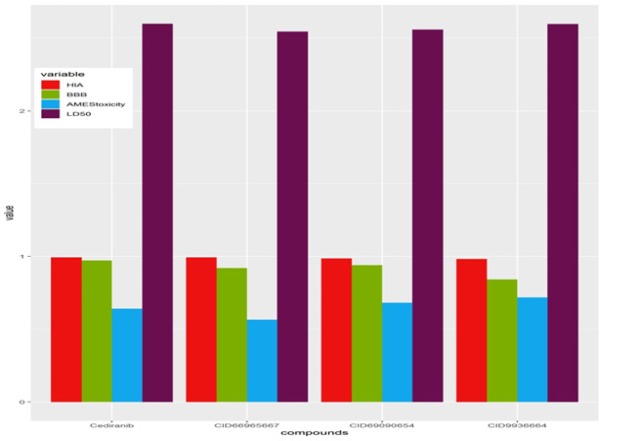
Comparative ADMET Studies of BBB, HIA, AMES Toxicity and LD50 of the Established Compounds (cediranib, PubID9936664) against Virtual Screened Compounds

**Figure 7:**
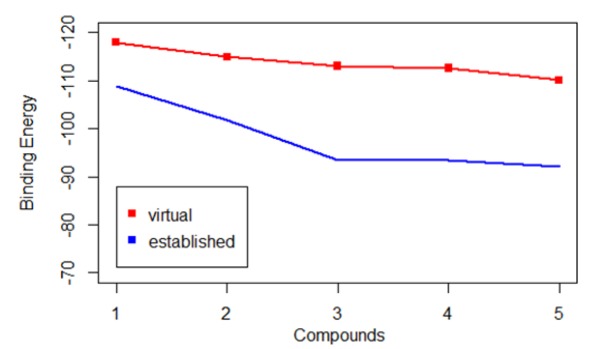
Drug-Likeness Study with Five Best Virtually Screened Compound and Five Best Established Inhibitors by Using OSIRIS Property Explorer Software

*Comparative ADMET profile study of the compounds and the control*

The comparative ADMET profile for the established and predicted inhibitors based on important parameters such as BBB (Blood-Brain Barrier), HIA (Human Intestinal Absorption), AMES toxicity, and LD50.Preferably four best chemical compounds, the best virtual screened inhibitor Pubchem ID-66965667 and established drug cediranib with the two other Pubchem CID:69090654 and Pubchem CID:9936664 has been taken for the comparative ADMET studies.

These four compounds were graphically projected using R-programming as revealed down in the (Figure 6). The parameters such as BBB, HIA, AMES Toxicity, and LD50 were obtained from the admetSAR database and were tabulated according to their properties and predicted values. As shown in the graph as well as tabular data (Table 7) among the four compounds the values of BBB were higher in the established drug cediranib and then the second best virtual screened compound with PubChem ID-69090654 as comparative to other compounds. Human Intestinal Absorption (HIA) of the best virtual drug (CID:66965667) compounds is somewhat higher than the cediranib. AMES toxicity is lowest for the virtual drug CID:69090654, among the best four Compounds. The LD50 is almost alike for all the four compounds.

*Drug-Likeness Prediction Studies*

Table 7 depicts the drug-likeness properties of two best virtually docked compounds and two best-established compounds using OSIRIS Property Explorer. Although clogP-value which stands for an algorithm of compound’s partition coefficient between N-octanol and water and molecular weight for an algorithm virtual screened compound (PubCID 69090654) have similar absorption property as the best established compound cediranib but log S values clarify that solubility of the best virtual screened compound (PubCID 66965667) was highest among best four. TPSA belonging to polar atoms in the compound showing less difference in the values of best virtually screened compound (PubCID 66965667) and the best-established drug cediranib. Beside all the properties, Figure 7 clearly shows the binding energy of the best virtual screened compound (PubCID 66965667) (-117.928) with the target *VEGF* was quite lesser than the best-established compound cediranib (-108.925) the property was proving that affinity of the newly screened compound for target protein *VEGF* in GBM was higher.

## Discussion

Being the most aggressive tumor in a human, Glioblastoma drew the noteworthy attention of scientists and researchers all over the world. According to the research in spite of having various treatments for glioblastoma including chemotherapy, radiation therapy, surgery, a diagnosed patient does not have long-term survival rate and also have numerous side effects (Nayarisseri et al., 2018; Sharda et al., 2019). In the present study, we proposed a newly screened inhibitor which directly can block the active site of VEGFR and have found to be less toxicity and cytotoxicity on ADMET analysis. Among the 16 established inhibitors from various studies that inhibit VEGF activity, Cediranib (Batchelor et al., 2007b), CEP-7055 (Jones-Bolin et al., 2006b) found to be best best known for the treatment of glioblastoma. In present study, we found an effective inhibitor SCHEMBL1250485 (PubChem CID: 66965667) by the mean of In silico-approaches.

The ranking and evaluation of the predicted inhibitor conformations is a critical aspect of structure-based virtual screening. However, estimations of structure-based screening parameters have yielded impressive results and numerous novel hits (Kitchen et al., 2004). Virtual screened inhibitor SCHEMBL1250485 have lower rerank score (-117.927) than the pre-established inhibitors which give the better affinity to the ligand to bind with the active site. SCHEMBL1250485 has a low probability of absorption for blood Brain barrier which is an essential property for a drug. Established inhibitor cediranib has a comparatively high chance for the same. SCHEMBL1250485 has low AMES toxicity as well as carcinogenicity than the best-established inhibitor cediranib. As SCHEMBL1250485 has better and enhanced properties as a suitable inhibitor of VEGFR in glioblastoma, our findings suggest the better active site inhibitor which can further be used for the In vitro studies and drug development.

In conclusion, Glioblastoma multiforme (GBM) is a fast-growing glioma that is developed from astrocytes and oligodendrocytes leads to devastating brain cancer, always leading to mortality within the span of a year. Vascular endothelial growth factor (*VEGF*) is the most abundant and essential mediator of angiogenesis in glioblastoma as it promotes proliferation. In this present research, an attempt has been made to demonstrate from the study of pre-existing inhibitors found against inhibitory protein *VEGF* which in turn help to inhibit Glioblastoma. The computational prediction of 16 pre-established drugs such as molecular docking studies followed by virtual screening and comparative studies of the best drugs from docking result and virtual docked result, we found the inhibitorCID:66965667has admirable properties to inhibit *VEGF*. Additionally, the best-established docked compound Cediranib (AZD2171) has PubChem CID:9933475 and the virtual screening resulted in compound PubChem CID:66965667 examine for ADMET study in terms of toxicity as a non-carcinogenic and non-mutagenic profile where both the perspectives reveal analogous properties, somehow PubChem CID:9933475 was found having higher probability in terms of BBB-Blood Brain Barrier, whereas HIA-Human Intestinal Absorption, AMES toxicity, and LD50 is having higher probability. In spite of radio-chemotherapy and advances in surgery, glioblastoma still shows a lot of resistance towards treatment and PubChem CID:66965667 medicines. In this scenario, new approaches to study glioblastoma and to design optimized therapies are greatly needed. The current study radicalised the potential inhibitor SCHEMBL1250485 endowed by ADMET and drug-likeness study with the enhanced binding affinity, therapeutic properties, less toxicity than established drug Cediranib. As per the comparative virtual screened drug properties with all prediction as a potential candidate, it could be also optimized for a remarkable pharmacological profile. Hence the study ahead on these compounds may be subjected to In vitro analysis for its pharmacokinetic and biological activity. 
